# On Chaos in the Fractional-Order Discrete-Time Unified System and Its Control Synchronization

**DOI:** 10.3390/e20070530

**Published:** 2018-07-15

**Authors:** Amina-Aicha Khennaoui, Adel Ouannas, Samir Bendoukha, Xiong Wang, Viet-Thanh Pham

**Affiliations:** 1 Department of Mathematics and Computer Sciences, University of Larbi Ben M’hidi, Oum El Bouaghi 04000, Algeria; 2 Department of Mathematics and Computer Science, University of Larbi Tebessi, Tebessa 12002, Algeria; 3 Electrical Engineering Department, College of Engineering at Yanbu, Taibah University, Medina 42353, Saudi Arabia; 4 Institute for Advanced Study, Shenzhen University, Shenzhen 518060, China; 5 Modeling Evolutionary Algorithms Simulation and Artificial Intelligence, Faculty of Electrical & Electronics Engineering, Ton Duc Thang University, Ho Chi Minh City, Vietnam; phamvietthanh@tdt.edu.vn

**Keywords:** fractional unified map, unified map, fractional discrete calculus, chaos control, chaos synchronization

## Abstract

In this paper, we propose a fractional map based on the integer-order unified map. The chaotic behavior of the proposed map is analyzed by means of bifurcations plots, and experimental bounds are placed on the parameters and fractional order. Different control laws are proposed to force the states to zero asymptotically and to achieve the complete synchronization of a pair of fractional unified maps with identical or nonidentical parameters. Numerical results are used throughout the paper to illustrate the findings.

## 1. Introduction

Chaotic dynamical systems have attracted a considerable level of attention over the last three decades due to the wide range of applications. Chaotic systems can be divided into two main categories: continuous-time and discrete-time. In this paper, we are interested in discrete-time chaotic systems, which are also referred to as chaotic maps. Over the years, a number of different chaotic maps have been proposed in the literature and applied in different fields including the Henon map [1], the Lozi system [2], the generalized Henon map [3], the Baier–Klein system [4], the Stefanski map [5], the Rössler map [6] and the Wang map [7]. Since the Henon and Lozi maps are the earliest discrete-time chaotic systems, they have been studied extensively by a vast number of researchers. One of the interesting studies is that of Zeraoulia and Sprott [8], where the authors proposed a new chaotic map as a combination of the the Henon and Lozi maps named the unified map. In this unified map, they included a parameter that can be varied between zero and one to alter the dynamics of the map with one end of the spectrum belonging to the Henon map and the other to the Lozi map. A summary of the unified map’s bifurcation and dynamics will be presented later on in Section 2.1.

The general idea of a fractional derivative for a continuous-time real function can be attributed to Gottfried Leibniz in a letter he wrote back in 1695. The proper definition of such a derivative, however, was not formulated until the late 19th Century as a result of the collective works of Liouville, Grunwald, Letnikov and Riemann. Fractional calculus has since been shown to be useful in the fields of capacitor theory, electrical circuits, chemistry, viscoelasticity, neurology, diffusion, control theory and statistics [9]. As for discrete-time functions, the first definition of a fractional difference operator was made by Diaz and Olser in 1974 [10]. The interesting thing about this operator is that it is a generalization of the binomial formula for the *n*-th difference operator by means of the Gamma function. The vast majority of literature related to fractional discrete calculus was published in the last decade including [11,12,13,14,15].

In recent years, since fractional discrete calculus became a subject of interest [15], focus has shifted towards fractional-order chaotic maps. To date, only a small number of fractional maps have been proposed in the literature [16,17,18,19], which has motivated the research presented in this paper. It is reported that these fractional chaotic maps have superior characteristics over their integer counterparts. In [19], the authors point out that the chaotic patterns exhibited by the fractional generalized Henon map depend on the fractional order. This means that the fractional map is more suitable for secure communications and encryption, as it includes a new degree of freedom. These added degrees of freedom can also be used in catching the hidden aspects of real-world phenomena encountered in ecology [18]. The author of [17] concluded that fractional maps have simpler forms, but hold richer dynamical behaviors than their integer counterparts.

In our study, we examine the fractional map corresponding to the unified discrete-time system and study its dynamics and control. Generally, when we talk about chaotic systems, we are interested in their control and synchronization. Control aims to adaptively force the chaotic states to a steady state, usually zero, [20,21], whereas synchronization is concerned with forcing a slave system to follow the same trajectory of a master with different initial conditions [22,23,24,25,26,27,28,29,30,31,32]. To the best of our knowledge, very few studies have been dedicated to the control and synchronization of fractional-order chaotic maps, including [33,34,35].

## 2. Results

Since the subject of fractional discrete calculus is still relatively new and the notation has not yet been settled, we start with a general description of the notation and stability results that will aid the reader in understanding the analysis to come. We note that similar to continuous-time fractional calculus, where numerous definitions exist for the fractional derivative of a function, the Caputo one is the most used today. Throughout this paper, the notation ${}^{C}\Delta_{a}^{\upsilon }X\left(t\right)$ is employed to denote the υ-Caputo type delta difference of a function $X\left(t\right):N_{a}\to R$ with $N_{a}=\left\{a,a+1,a+2,\ldots \right\}$ [12] defined as:

(1) $${}^{C}\Delta_{a}^{\upsilon }X\left(t\right)=\Delta_{a}^{-(n-\upsilon )}\Delta^{n}X\left(t\right)=\frac{1}{\Gamma \left(n-\upsilon \right)}\sum_{s=a}^{t-\left(n-\upsilon \right)}{\left(t-\sigma \left(s\right)\right)}^{\left(n-\upsilon -1\right)}\Delta_{s}^{n}X\left(s\right),$$

where υ∉N is the fractional order, $t\in N_{a+n-\upsilon }$, and $n=\left[\upsilon \right]+1$. In (11), the υ-th fractional sum of $\Delta_{s}^{n}X\left(t\right)$ is defined similar to [11] as:

(2) $$\Delta_{a}^{-\upsilon }X\left(t\right)=\frac{1}{\Gamma \left(\upsilon \right)}\sum_{s=a}^{t-\upsilon }{\left(t-\sigma \left(s\right)\right)}^{\left(\upsilon -1\right)}X\left(s\right),$$

with υ>0, σ(s)=s+1. The term $t^{\upsilon }$ denotes the falling function defined in terms of the Gamma function Γ as:

(3) $$t^{\upsilon }=\frac{\Gamma \left(t+1\right)}{\Gamma \left(t+1-\upsilon \right)}.$$

The following two theorems describe the numerical formula for a fractional discrete map, as well as the fractional discrete direct Lyapunov method, respectively. In order to use Theorem 2 later on, we will require the inequality described by Lemma 1 below.

**Theorem** **1.** *[36] For the delta fractional difference equation:*

(4) $$\left\{\begin{array}{c} {}^{C}\Delta_{a}^{\upsilon }u\left(t\right)=f\left(t+\upsilon -1,u\left(t+\upsilon -1\right)\right),\\ \Delta^{k}=u_{k},\;n=\left[\upsilon \right]+1,\;k=0,1,\ldots ,n-1 \end{array}\right.$$

*the equivalent discrete integral equation can be obtained as:*

(5) $$u\left(t\right)=u_{0}\left(t\right)+\frac{1}{\Gamma (\upsilon )}\sum_{s=a+n-\upsilon }^{t-\upsilon }{\left(t-\sigma \left(s\right)\right)}^{\left(\upsilon -1\right)}f\left(s+\upsilon -1,u\left(s+\upsilon -1\right)\right),t\in N_{\alpha +n},$$

*where:*

(6) $$u_{0}\left(t\right)=\sum_{k=0}^{m-1}\frac{{\left(t-a\right)}^{k}}{k}\Delta^{k}u\left(a\right).$$

**Theorem** **2.** *[14] If there exists a positive definite Lyapunov function $V\left(X\left(t\right)\right)$ such that:*

(7) $${}^{C}\Delta_{a}^{\upsilon }V\left(X\left(t\right)\right)<0\;\mathrm{for}\;\mathrm{all}\;t\in N_{a+1-\upsilon },$$

*then the trivial solution of the system is asymptotically stable.*

**Lemma** **1.** *[14] For $X\left(t\right)={\left(x_{1}\left(t\right),\ldots ,x_{n}\left(t\right)\right)}^{T}$, 0<υ≤1 and $\forall t\in N_{a+1-\upsilon }$, the following inequality holds:*

(8) $$\frac{1}{2}{\;}^{C}\Delta_{a}^{\upsilon }\left(X^{T}\left(t\right)X\left(t\right)\right)\leq X^{T}\left(t+\upsilon -1\right){\;}^{C}\Delta_{a}^{\upsilon }X\left(t\right).$$

The following subsections describe the main findings of our study. Section 2.1 describes the signal model of the proposed fractional unified map and investigates its entropy and chaotic dynamics by means of phase portraits and bifurcation plots. Section 2.2 and Section 2.3 describe the proposed stabilization and synchronization schemes.

### 2.1. Chaos in the Fractional-Order Unified Map

In [8], Zeraoulia and Sprott proposed a new unified piecewise chaotic map:

(9) $$\left\{\begin{array}{c} x\left(n+1\right)=1-1.4f_{\alpha }\left(x\left(n\right)\right)+y\left(n\right),\\ y\left(n+1\right)=0.3x\left(n\right) \end{array}\right.$$

where the bifurcation parameter α is in the range 0≤α≤1 and function $f_{\alpha }$ is defined as:

(10) $$f_{\alpha }\left(x\left(n\right)\right)=\alpha \left|x\left(n\right)\right|+\left(1-\alpha \right)x^{2}\left(n\right).$$

**Figure 1:**
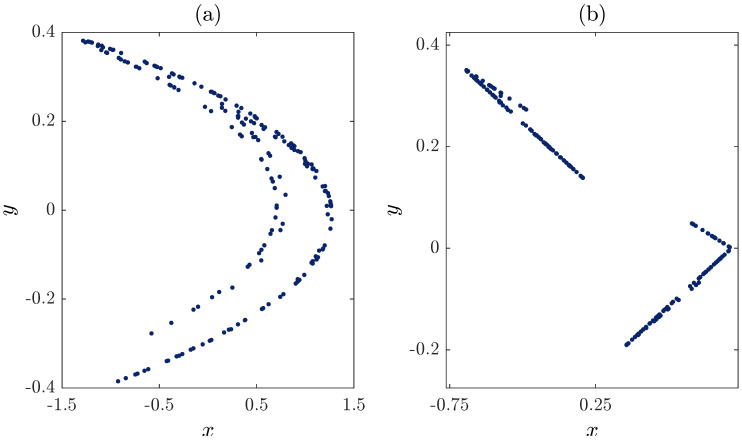
(**a**) The original Henon chaotic attractor obtained from the unified chaotic map for α=0. (**b**) The original Lozi chaotic attractor obtained from the unified chaotic map for α=1.

The importance of this unified map stems from the fact that setting α to zero yields the classical Hénon map depicted in Figure 1a and given by:

(11) $$\left\{\begin{array}{c} x\left(n+1\right)=1-1.4x^{2}\left(n\right)+y\left(n\right),\\ y\left(n+1\right)=0.3x\left(n\right). \end{array}\right.$$

On the other hand, when α=1, we end up with the classical Lozi map depicted in Figure 1b and defined as:

(12) $$\left\{\begin{array}{c} x\left(n+1\right)=1-1.4\left|x\left(n\right)\right|+y\left(n\right),\\ y\left(n+1\right)=0.3x\left(n\right). \end{array}\right.$$

**Figure 2:**
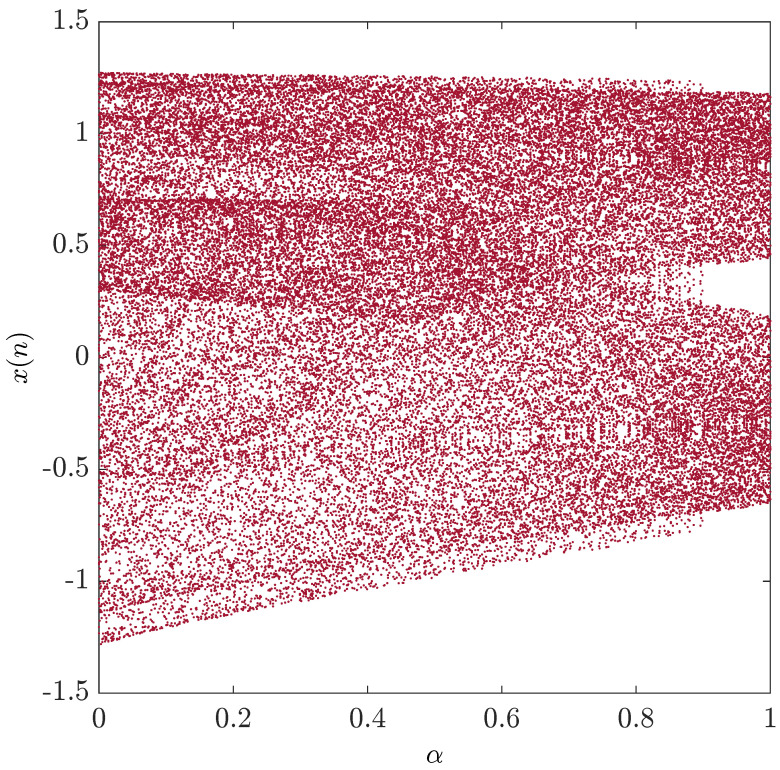
The bifurcation diagram of the unified chaotic map with the critical parameter 0≤α≤1 being changed in steps of Δα=0.001.

What is more interesting is that they demonstrated how the system exhibits a chaotic behavior for 0≤α≤1. Figure 2 shows the bifurcation diagram with the critical parameter α being changed in steps of Δα=0.001. System (9) may be rewritten in the form:

(13) $$\left\{\begin{array}{c} \Delta x\left(n\right)=1-1.4f_{\alpha }\left(x\left(n\right)\right)+y\left(n\right)-x\left(n\right),\\ \Delta y\left(n\right)=0.3x\left(n\right)-y\left(n\right). \end{array}\right.$$

Then, using the discrete fractional calculus described in the previous section, we may define the fractional unified map as:

(14) $$\left\{\begin{array}{c} {}^{C}\Delta_{a}^{\upsilon }x\left(t\right)=1-1.4f_{\alpha }\left(x\left(t-1+\upsilon \right)\right)+y\left(t-1+\upsilon \right)-x\left(t-1+\upsilon \right),\\ {}^{C}\Delta_{a}^{\upsilon }y\left(t\right)=0.3x\left(t-1+\upsilon \right)-y\left(t-1+\upsilon \right), \end{array}\right.$$

for $t\in N_{a+1-\upsilon }$, where ${}^{C}\Delta_{a}^{\upsilon }x\left(t\right)$ and ${}^{C}\Delta_{a}^{\upsilon }y\left(t\right)$ are the Caputo-like delta differences of states $x\left(t\right)$ and $y\left(t\right)$, respectively, and 0<υ≤1 is the fractional order.

Following Theorem 2, using the discrete kernel function:

(15) $${\left(t-\sigma \left(s\right)\right)}^{\upsilon -1}=\frac{\Gamma (t-s)}{\Gamma (t-s-\upsilon +1)},$$

and assuming that a=0, the numerical formulas for the fractional map (14) may be obtained as:

(16) $$\left\{ \begin{array}{cc} x\left(n\right) & =x\left(0\right)+\frac{1}{\Gamma \left(\upsilon \right)}\sum_{j=1}^{n}\frac{\Gamma (n-j+\upsilon )}{\Gamma (n-j+1)}\\ & \;\left(1-1.4f_{\alpha }\left(x\left(j-1\right)\right)+y\left(j-1\right)-x\left(j-1\right)\right),\\ y\left(n\right) & =y\left(0\right)+\frac{1}{\Gamma \left(\upsilon \right)}\sum_{j=1}^{n}\frac{\Gamma \left(n-j+\upsilon \right)}{\Gamma \left(n-j+1\right)}\left(0.3x\left(j-1\right)-y\left(j-1\right)\right). \end{array}\right.$$

**Figure 3:**
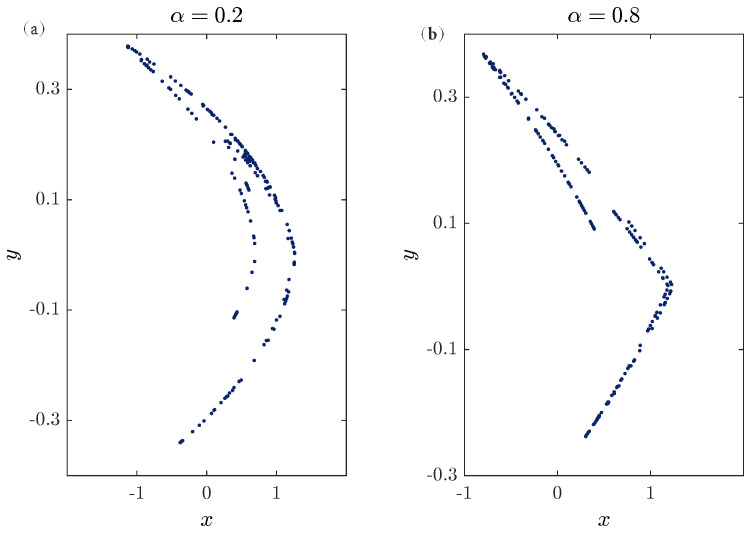
(**a**) A chaotic attractor obtained from the unified chaotic map for α=0.2. (**b**) A chaotic attractor obtained from the unified chaotic map for α=0.8.

**Figure 4:**
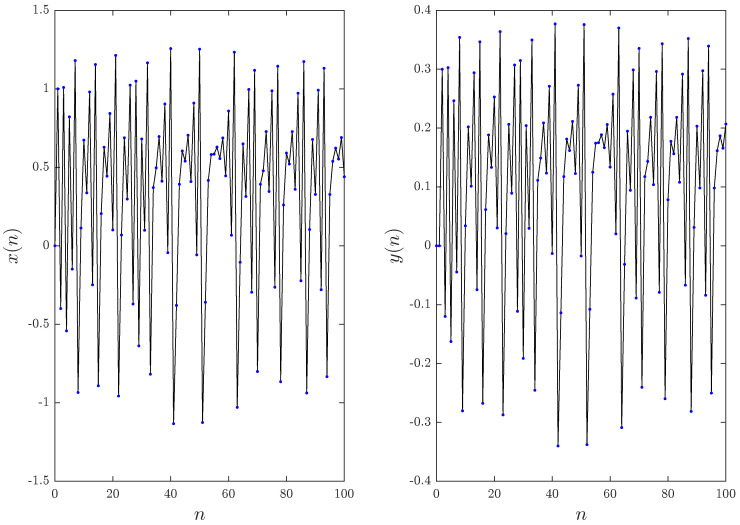
Time evolution of the states for the fractional the unified chaotic map for υ=1 and α=0.2.

**Figure 5:**
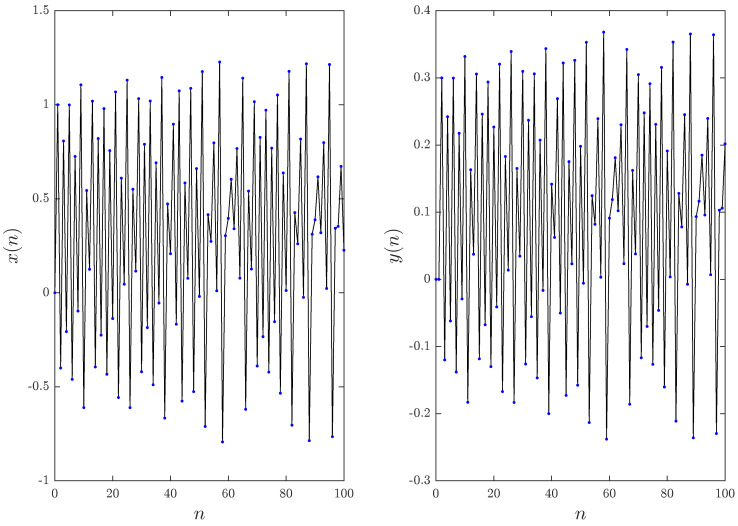
Time evolution of the states for the fractional unified chaotic map for υ=1 and α=0.8.

In [8], it was reported that while the bifurcation parameter α is close to zero, the function $f_{\alpha }$ defined in (10) behaves similar to the term $x^{2}\left(n\right)$. and when α is close to one it behaves similar to the absolute function $\left|x\left(n\right)\right|$. Since the values α=0 and α=1 belong to the fractional Hénon and Lozi maps, which have been studied previously in the literature, we choose to ignore them and investigated the numerical Formula (16) over the range 0<α<1. Considering the values α=0.8 and α=0.2, Figure 3 depicts the phase space for n=200 and υ=1 with initial conditions $x\left(0\right)=y\left(0\right)=0$. The states of the fractional map for 100 points are displayed in Figure 4 and Figure 5. Observe that in this case, the fractional map (14) refers to the classical map.

**Figure 6:**
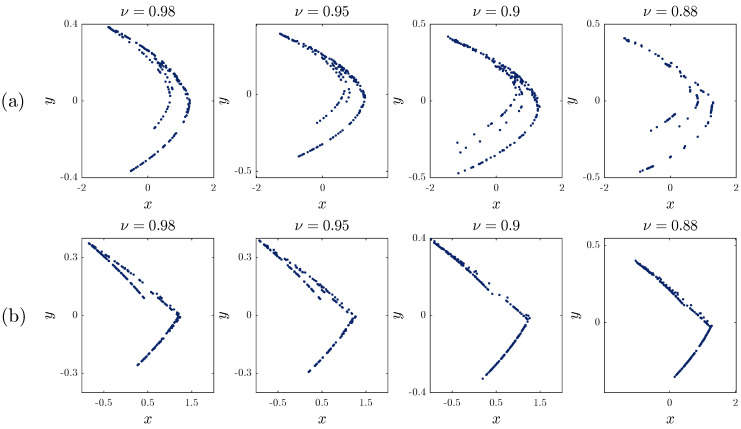
Phase portraits of the fractional unified map for: (**a**) α=0.2 and (**b**) α=0.8 with different fractional orders.

With the same parameters and the same initial condition, using numerical Formula (16), the fractional map is shown in Figure 6 for different fractional orders υ. As υ decreases, the phase plane of the fractional maps changes its shape until it completely disappears. For α=0.2, the minimum fractional order that produced a bounded attractor is υ=0.88. On the other hand, when α=0.8, we see that we obtain a bounded attractor as far as υ=0.4.

The “approximate entropy” was proposed by Pincus to present the complexity of time series [37,38]. Approximate entropy measurement is helpful and applied to discover different chaotic systems [39,40]. The brief computation of the approximate entropy is presented as follows. By selecting a sequence of *N* data samples $s\left(1\right),s\left(2\right),\ldots ,s\left(N\right)$, a sequence of vectors is constructed:

(17) $$S\left(i\right)=\left[s\left(i\right),s\left(i+1\right),\ldots ,s\left(i+m-1\right)\right]\;\mathrm{with}\;1\leq i\leq N-m+1,$$

in which *m* is the embedding dimension. The distance between the vector $S\left(i\right)$ and the vector $S\left(j\right)$ is $d\left(S\left(i\right),S\left(j\right)\right)$. It is noted that we choose the following threshold [37,38]:

(18) $$r=0.2\mathrm{std}\left(s\right),$$

where std$\left(s\right)$ denotes the standard deviation of the data *s*. As a result, we can calculate the number of vectors (*K*) having distance $d\left(S\left(i\right),S\left(j\right)\right)\leq r$ [37,38]. The approximate entropy is:

(19) $$\mathrm{ApEn}=\phi^{m}\left(r\right)-\phi^{m+1}\left(r\right),$$

in which $\phi^{m}\left(r\right)$ is given by:

(20) $$\phi^{m}\left(r\right)=\frac{1}{N-m-1}\sum_{i=1}^{N-m+1}\log C_{i}^{m}\left(r\right),$$

and $C_{i}^{m}\left(r\right)$ is defined by:

(21) $$C_{i}^{m}\left(r\right)=\frac{K}{N-m+1}.$$

We have calculated the approximate entropy (ApEn) for the fractional-order unified map, and the results are reported in Table 1. For α=0.2, the complexity of the fractional-order unified map is reduced when the value of υ is decreased to 0.88. For α=0.8, when reducing the value of υ from 0.98 to 0.88, the complexity of the map varies. The results display the changes of the phase portraits in Figure 6.

**Figure 7:**
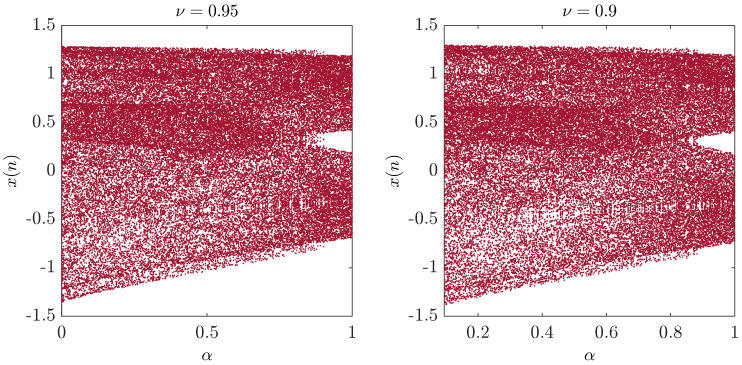
The bifurcation diagram of the unified chaotic map with α as the critical parameter for υ=0.95 and υ=0.9.

**Figure 8:**
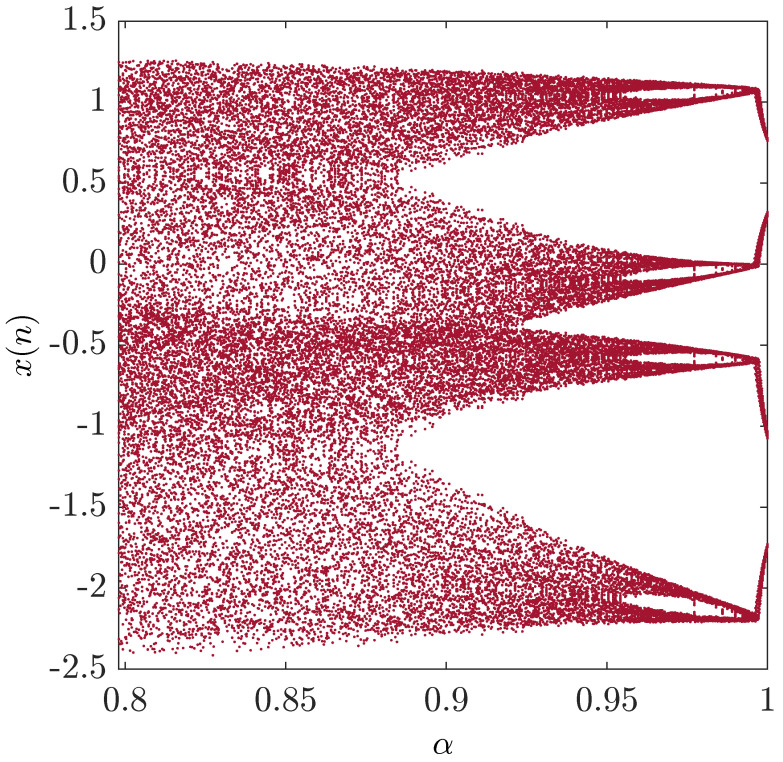
The bifurcation diagram of the fractional unified chaotic map for υ=0.4 with α as the critical parameter.

**Figure 9:**
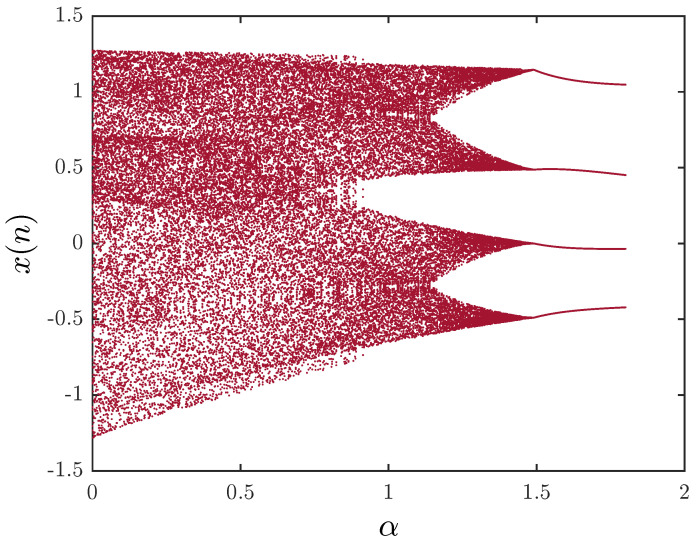
Bifurcation diagram of the fractional unified chaotic map for υ=0.9 with $\alpha \in \left[0,1.8\right]$.

To investigate the chaotic behavior of the fractional map, we study the bifurcation of the parameter α with the step size Δα=0.001. Figure 7 illustrates the results. The figure demonstrates clearly the chaotic behavior of the fractional map. When υ=1, chaos is observed in the interval [0,1]. A decrease in the fractional order υ leads to a decrease in the interval, where chaos is apparent. As shown in Figure 8, for υ=0.4, chaos is seen in the interval $\alpha \in \left[0.799,0.95\right]$. A better understanding of the route to chaos can be seen in Figure 9, where the range of α is increased to 1.8. However, for $\alpha \in \left[0,1.8\right]$, a border-collision bifurcation scenario is observed. The map begins with a fully-developed chaotic regime, and increasing α leads to the disappearance of the chaotic band and the appearance of a four-period orbit. Experiments have also shown that even with fractional orders υ less than one, the fractional map still behaves in a similar manner to the standard case with the one exception that the chaotic interval varies with υ.

### 2.2. Control of the Fractional-Order Unified Map

In this section, we are interested in a one-dimensional adaptive control law that forces the states of the proposed two-dimensional fractional unified system to zero asymptotically. Chaos control is an important subject, particularly in engineering, as it may have applications in stabilizing a chaotic dynamical system, e.g., a two degree of freedom robot arm.

**Theorem** **3.** *The 2D fractional-order unified chaotic map can be controlled under the 1D control law:*

(22) $$U=1.4f_{\alpha }\left(x\left(t\right)\right)-1.3y\left(t\right)-1.$$

**Proof.**  The controlled fractional-order unified chaotic map can be described as follows:

(23) $$\left\{\begin{array}{c} {}^{C}\Delta_{a}^{\upsilon }x\left(t\right)=1-1.4f_{\alpha }\left(x\left(t-1+\upsilon \right)\right)+y\left(t-1+\upsilon \right)-x\left(t-1+\upsilon \right)+U,\\ {}^{C}\Delta_{a}^{\upsilon }y\left(t\right)=0.3x\left(t-1+\upsilon \right)-y\left(t-1+\upsilon \right). \end{array}\right.$$

Substituting the proposed control law (22) in (23) leads to the new dynamics:

(24) $$\left\{\begin{array}{c} {}^{C}\Delta_{a}^{\upsilon }x\left(t\right)=-x\left(t-1+\upsilon \right)-0.3y\left(t-1+\upsilon \right),\\ {}^{C}\Delta_{a}^{\upsilon }y\left(t\right)=0.3x\left(t-1+\upsilon \right)-y\left(t-1+\upsilon \right). \end{array}\right.$$

Since the aim of the control law (22) is to force the two states towards zero asymptotically, what we want to do is to show that the zero solution of this resulting controlled system dynamics is globally asymptotically stable. In order to do so, we employ the fractional discrete Lyapunov method described in Theorem 2. We propose the Lyapunov function:

(25) $$V\left(t\right)=\frac{1}{2}\left(x^{2}\left(t\right)+y^{2}\left(t\right)\right),$$

leading to:

(26) $${}^{C}\Delta_{a}^{\upsilon }V\left(t\right)=\frac{1}{2}\left({\;}^{C}\Delta_{a}^{\upsilon }x^{2}\left(t\right)+{\;}^{C}\Delta_{a}^{\upsilon }y^{2}\left(t\right)\right).$$

Using the inequality of Lemma 1, we see that:

(27) $$\begin{array}{cc} {}^{C}\Delta_{a}^{\upsilon }V\left(t\right) & \leq x\left(t-1+\upsilon \right){\;}^{C}\Delta_{a}^{\upsilon }x\left(t\right)+y\left(t-1+\upsilon \right){\;}^{C}\Delta_{a}^{\upsilon }y\left(t\right)\\ & =-\left(x^{2}\left(t-1+\upsilon \right)+y^{2}\left(t-1+\upsilon \right)\right)<0. \end{array}$$

Therefore, by Theorem 2, we conclude that the zero solution of System (23) is in fact globally asymptotically stable, and thus, the fractional unified map is controlled. ☐

**Figure 10:**
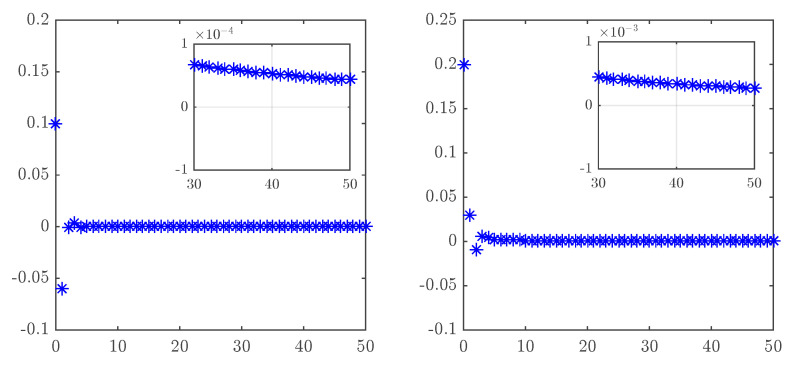
Time evolution of the controlled states of the fractional unified map with υ=0.95, α=0.2 and a=0.

In order to put Theorem 3 to the test, a MATLAB script was run taking υ=0.95 and a=0 and following the time evolution of the states of (23). The result in Figure 10 clearly shows how the states progress towards zero.

### 2.3. Synchronization

Perhaps synchronization is the most interesting aspect of chaotic dynamical systems in general, as it allows many applications of chaos. In this section, we present two synchronization schemes related to the proposed fractional unified system. The first deals with two fractional identical unified maps and the second with different maps. The terms identical and different in this context refer to the parameter α having the same values or different values, respectively.

#### 2.3.1. Synchronization of Identical Fractional Unified Maps

Let us consider the master system described for ${t\in N}_{a+1-\upsilon }$ by:

(28) $$\left\{\begin{array}{cc} {}^{C}\Delta_{a}^{\upsilon }x_{m}\left(t\right) & =-1.4\left(\alpha \left|x_{m}\left(t-1+\upsilon \right)\right|+\left(1-\alpha \right)x_{m}^{2}\left(t-1+\upsilon \right)\right)\\ & \;+y_{m}\left(t-1+\upsilon \right)+1-x_{m}\left(t-1+\upsilon \right),\\ {}^{C}\Delta_{a}^{\upsilon }y_{m}\left(t\right) & =0.3x_{m}\left(t-1+\upsilon \right)-y_{m}\left(t-1+\upsilon \right). \end{array}\right..$$

Note that the subscript *m* in the states refers to the master. As for the slave system, we choose the exact same map, but use a subscript *s* instead and with the addition of a controller for the first state, i.e.,:

(29) $$\left\{\begin{array}{cc} {}^{C}\Delta_{a}^{\upsilon }x_{s}\left(t\right) & =-1.4\left(\alpha \left|x_{s}\left(t-1+\upsilon \right)\right|+\left(1-\alpha \right)x_{s}^{2}\left(t-1+\upsilon \right)\right)\\ & \;+y_{s}\left(t-1+\upsilon \right)+1-x_{s}\left(t-1+\upsilon \right)+u,\\ {}^{C}\Delta_{a}^{\upsilon }y_{s}\left(t\right) & =0.3x_{s}\left(t-1+\upsilon \right)-y_{s}\left(t-1+\upsilon \right), \end{array}\right.$$

where *u* is a controller to be determined later. The aim of synchronization is to force the error system:

(30) $$\left\{\begin{array}{c} e_{1}\left(t\right)=x_{s}\left(t\right)-x_{m}\left(t\right),\\ e_{2}\left(t\right)=y_{s}\left(t\right)-y_{s}\left(t\right). \end{array}\right.$$

to zero asymptotically. The following theorem presents the proposed control law.

**Theorem** **4.** *Subject to:*

(31) $$u=1.4\alpha \left(\left|x_{s}\left(t\right)\right|-\left|x_{m}\left(t\right)\right|\right)-1.3e_{2}\left(t\right)-2.8l\left(1+\alpha \right)e_{1}\left(t\right)$$

*where $\left|x_{s}\left(t\right)\right|=\left|x_{m}\left(t\right)\right|\leq l$, the master and slave pair is globally synchronized.*

**Proof.**  The fractional difference equations related to error System (30) can be given by:

(32) $$\left\{\begin{array}{cc} {}^{C}\Delta_{a}^{\upsilon }e_{1} & =-1.4\left[\alpha \left|x_{s}\left(t-1+\upsilon \right)\right|+\left(1-\alpha \right)x_{s}^{2}\left(t-1+\upsilon \right)\right]+y_{s}\left(t-1+\upsilon \right)\\ & \;+1-x_{s}\left(t-1+\upsilon \right)+1.4\left[\alpha \left|x_{m}\left(t-1+\upsilon \right)\right|+\left(1-\alpha \right)\right.\\ & \;\left.x_{m}^{2}\left(t-1+\upsilon \right)\right]-y_{m}\left(t-1+\upsilon \right)-1+x_{m}\left(t-1+\upsilon \right)+u\\ & =1.4\alpha \left(\left|x_{m}\left(t-1+\upsilon \right)\right|-\left|x_{s}\left(t-1+\upsilon \right)\right|\right)-1.4\left(1-\alpha \right)\\ & \;\left(x_{s}\left(t-1+\upsilon \right)+x_{m}\left(t-1+\upsilon \right)\right)e_{1}+e_{2}-e_{1}+u,\\ {}^{C}\Delta_{a}^{\upsilon }e_{2} & =0.3x_{s}\left(t-1+\upsilon \right)-y_{s}\left(t-1+\upsilon \right)-0.3x_{m}\left(t-1+\upsilon \right)\\ & \;+y_{m}\left(t-1+\upsilon \right)\\ & =0.3e_{1}-e_{2}. \end{array}\right.$$

Substituting the control law (31) yields the simplified system:

(33) $$\left\{\begin{array}{cc} {}^{C}\Delta_{a}^{\upsilon }e_{1} & =-1.4\left(1-\alpha \right)\left(x_{s}\left(t-1+\upsilon \right)+x_{m}\left(t-1+\upsilon \right)\right)e_{1}\\ & \;-2.8l\left(1+\alpha \right)e_{1}-0.3e_{2},\\ {}^{C}\Delta_{a}^{\upsilon }e_{2} & =0.3e_{1}-e_{2}. \end{array}\right.$$

Using the same Lyapunov functional from Theorem 3 and employing Lemma 1, we have:

(34) $$\begin{array}{cc} {}^{C}\Delta_{a}^{\upsilon }V\left(t\right) & =\frac{1}{2}{\;}^{C}\Delta_{a}^{\upsilon }e_{1}^{2}\left(t\right)+\frac{1}{2}{\;}^{C}\Delta_{a}^{\upsilon }e_{2}^{2}\left(t\right)\\ & \leq e_{1}\left(t-1+\upsilon \right){\;}^{C}\Delta_{a}^{\upsilon }e_{1}\left(t\right)+e_{2}\left(t-1+\upsilon \right){\;}^{C}\Delta_{a}^{\upsilon }e_{2}\left(t\right)\\ & \leq -1.4\left(1-\alpha \right)\left[x_{s}\left(t-1+\upsilon \right)+x_{m}\left(t-1+\upsilon \right)\right]e_{1}^{2}\\ & \;-\left(2.8l\left(1+\alpha \right)+1\right)e_{1}^{2}-0.3e_{1}e_{2}+0.3e_{2}e_{1}-e_{2}^{2}\\ & \leq 1.4\left(1+\alpha \right)\left(\left|x_{s}\left(t-1+\upsilon \right)\right|+\left|x_{m}\left(t-1+\upsilon \right)\right|\right)e_{1}^{2}\\ & \;-\left(2.8l\left(1+\alpha \right)+1\right)e_{1}^{2}-e_{2}^{2}\\ & \leq 2.8l\left(1+\alpha \right)e_{1}^{2}-\left(2.8l\left(1+\alpha \right)+1\right)e_{1}^{2}-e_{2}^{2}\\ & =-\left(e_{1}^{2}+e_{2}^{2}\right)<0. \end{array}$$

It then follows directly from Theorem 2 that the zero solution of error System (33) is globally asymptotically stable. Hence, regardless of the initial conditions, the errors are guaranteed to converge towards zero asymptotically, meaning that the master and slave maps are synchronized. ☐

**Figure 11:**
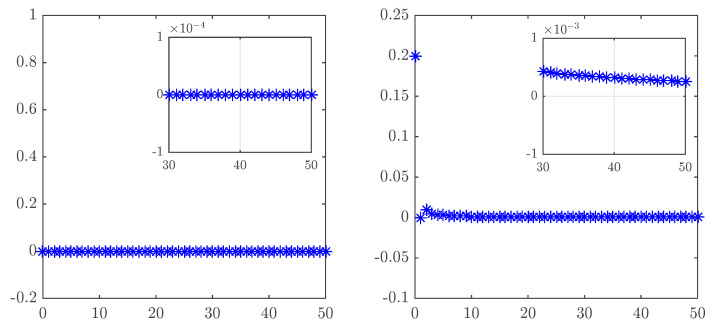
Time evolution of the synchronization errors for the identical fractional-order unified chaotic maps with υ=0.95, α=0.2 and a=0.

The control law (31) was implemented in MATLAB with υ=0.95 and a=0. Over time, the synchronization error depicted in Figure 11 can be seen to converge towards zero.

#### 2.3.2. Synchronization of Different Fractional Unified Maps

Let us, now, consider master System (28) with the slave:

(35) $$\left\{\begin{array}{cc} {}^{C}\Delta_{a}^{\upsilon }x_{s}\left(t\right) & =-1.4\left(\beta \left|x_{s}\left(t-1+\upsilon \right)\right|+\left(1-\beta \right)x_{s}^{2}\left(t-1+\upsilon \right)\right)\\ & \;+y_{s}\left(t-1+\upsilon \right)+1-x_{s}\left(t-1+\upsilon \right)+u,\\ {}^{C}\Delta_{a}^{\upsilon }y_{s}\left(t\right) & =0.3x_{s}\left(t-1+\upsilon \right)-y_{s}\left(t-1+\upsilon \right), \end{array}\right.$$

for ${t\in N}_{a+1-\upsilon }$ and with β≠α. The error system is defined in the same way as in (30). Theorem 5 below presents the one-dimensional control law that will generally synchronize the slave to the master regardless of the values of α and β.

**Theorem** **5.** *Subject to*

(36) $$\begin{array}{ccc} u & = & -1.4\left[\alpha \left|x_{s}\left(t-1+\upsilon \right)\right|+\left(1-\alpha \right)x_{s}^{2}\left(t-1+\upsilon \right)\right]\\ & & +1.4\left[\beta \left|x_{m}\left(t-1+\upsilon \right)\right|+\left(1-\beta \right)x_{m}^{2}\left(t-1+\upsilon \right)\right]-1.3e_{2}, \end{array}$$

*the master-slave pair (28)–(35) is synchronized.*

**Proof.**  This result can be proven in much the same way as Theorem 4 was using the same Lyapunov function and the error dynamics:

(37) $$\left\{\begin{array}{cc} {}^{C}\Delta_{a}^{\upsilon }e_{1} & =-1.4\left[\alpha \left|x_{s}\left(t-1+\upsilon \right)\right|+\left(1-\alpha \right)x_{s}^{2}\left(t-1+\upsilon \right)\right]\\ & \;+y_{s}\left(t-1+\upsilon \right)+1-x_{s}\left(t-1+\upsilon \right)+1.4\left[\beta \left|x_{m}\left(t-1+\upsilon \right)\right|\right.\\ & \;\left.+\left(1-\beta \right)x_{m}^{2}\left(t-1+\upsilon \right)\right]-y_{m}\left(t-1+\upsilon \right)-1+x_{m}\left(t-1+\upsilon \right)+u,\\ {}^{C}\Delta_{a}^{\upsilon }e_{2} & =0.3x_{s}\left(t-1+\upsilon \right)-y_{s}\left(t-1+\upsilon \right)-0.3x_{m}\left(t-1+\upsilon \right)\\ & \;+y_{m}\left(t-1+\upsilon \right), \end{array}\right.$$

leading to:

(38) $$\left\{\begin{array}{c} {}^{C}\Delta_{a}^{\upsilon }e_{1}=-e_{1}-0.3e_{2},\\ {}^{C}\Delta_{a}^{\upsilon }e_{2}=0.3e_{1}-e_{2}. \end{array}\right.$$

Using the same previous Lyapunov function, we have:

(39) $$\begin{array}{cc} {}^{C}\Delta_{a}^{\upsilon }V\left(t\right) & =\frac{1}{2}{\;}^{C}\Delta_{a}^{\upsilon }e_{1}^{2}\left(t\right)+\frac{1}{2}{\;}^{C}\Delta_{a}^{\upsilon }e_{2}^{2}\left(t\right)\\ & \leq e_{1}\left(t-1+\upsilon \right){\;}^{C}\Delta_{a}^{\upsilon }e_{1}\left(t\right)+e_{2}\left(t-1+\upsilon \right){\;}^{C}\Delta_{a}^{\upsilon }e_{2}\left(t\right)\\ & \leq -e_{1}^{2}-e_{2}^{2}<0. \end{array}$$

Again, it is easy to conclude that the zero error of (38) is globally asymptotically stable and that the master and slave are synchronized. ☐

**Figure 12:**
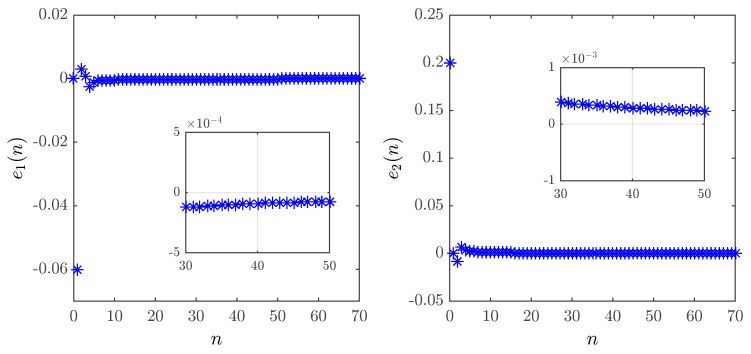
Time evolution of the synchronization errors for the different fractional-order unified chaotic maps with υ=0.95 and a=0.

Figure 12 shows the time evolution of the synchronization errors for the fractional maps with different parameters. We assumed a=0 and υ=0.95. Again, the errors can be easily shown to converge towards zero asymptotically.

## 3. Discussion

In this paper, we have proposed a fractional-order unified map based on the integer-order unified map developed by Zeraoulia and Sprott in [8] as a cross-over system between the Hénon and Lozi maps. The dynamics and bifurcations of the proposed map are discussed. Based on the results, we see that the bifurcation parameter α has an effect on the range of fractional orders for which chaos is observed. The lower α is, the lower the range becomes. In addition, we see that the fractional order also has an impact on the existence and shape of the chaotic behavior. This is what was referred to in [18] as an added degree of freedom. For instance, if the map is to be used in a secure communications or encryption setting, then it will allow for a wider range of pseudo-random keys. We have also calculated and presented the approximate entropy values of the fractional-order map for various values of the fractional order. Again, we see that the values of α and υ both have an impact on the entropy.

This paper also proposed a one-dimensional adaptive control strategy that forces the states towards zero asymptotically. The convergence of the states to zero was established by means of the Lyapunov method and verified by means of numerical results. Furthermore, two one-dimensional synchronization schemes were proposed for master and slave fractional unified maps with identical or nonidentical parameters. Numerical results were presented to confirm the success of these synchronization schemes.

## Figures and Tables

**Table 1:** Approximate entropy calculation of the fractional-order unified map for different fractional orders.

α	υ	**ApEn**	α	υ	**ApEn**
0.2	0.98	0.4037	0.8	0.98	0.2451
0.2	0.95	0.4511	0.8	0.95	0.2571
0.2	0.90	0.4407	0.8	0.90	0.2304
0.2	0.88	0.0981	0.8	0.88	0.2530
